# Supplementary material for: Controllable Synthesis, Formation Mechanism, and Photocatalytic Activity of Tellurium with Various Nanostructures
Source: Micromachines (Basel). 2023 Dec 19;15(1):1. doi: 10.3390/mi15010001 (PMC10818636; doi:10.3390/mi15010001)
Supplement: Supplementary file 1 [file micromachines-15-00001-s001.zip › micromachines-2762252-supplementary.pdf]

Supplementary Materials

Controllable Synthesis, Formation Mechanism, and Photocatalytic Activity of Tellurium with Various Nanostructures

Huan Wang [1,2,†], Hanlin Zou [2,†], Chao Wang [1], Sa Lv [1], Yujie Jin [2], Hongliang Hu [2], Xinwei Wang [3,*], Yaodan Chi [1] and Xiaotian Yang [4,*]

[1] Key Laboratory for Comprehensive Energy Saving of Cold Regions Architecture of Ministry of Education, Jilin Jianzhu University, Changchun 130118, China; wanghuan@jlju.edu.cn (H.W.); wangchao@jlju.edu.cn (C.W.); lvsa82@163.com (S.L.); chiyaodan@jlju.edu.cn (Y.C.)
[2] Department of Materials Science, Jilin Jianzhu University, Changchun 130118, China; 19819830942@163.com (H.Z.); jinyujie@jlju.edu.cn (Y.J.); huhongliang@126.com (H.H.)
[3] Engineering Research Center of Optoelectronic Functional Materials, Ministry of Education, School of Materials Science and Engineering, Changchun University of Science and Technology, Changchun 130022, China
[4] Department of Chemistry, Jilin Normal University, Siping 136000, China
* Correspondence: wxw4122@cust.edu.cn (X.W.); hanyxt@163.com (X.Y.)
† These authors contributed equally to this work.

S.II. The Pseudo First-Order Kinetic Equation

The pseudo first-order kinetic equation, $\ln (C_0/C_t) = kt$, is the reaction chemical reaction rate with the reaction or reaction related to the conditions (concentration) of the kinetic equation, where C_0 is the initial concentration, C_t is the concentration at time t, and k is the apparent rate constant. The kinetics of the degradation reaction can be described by the first order kinetic model.

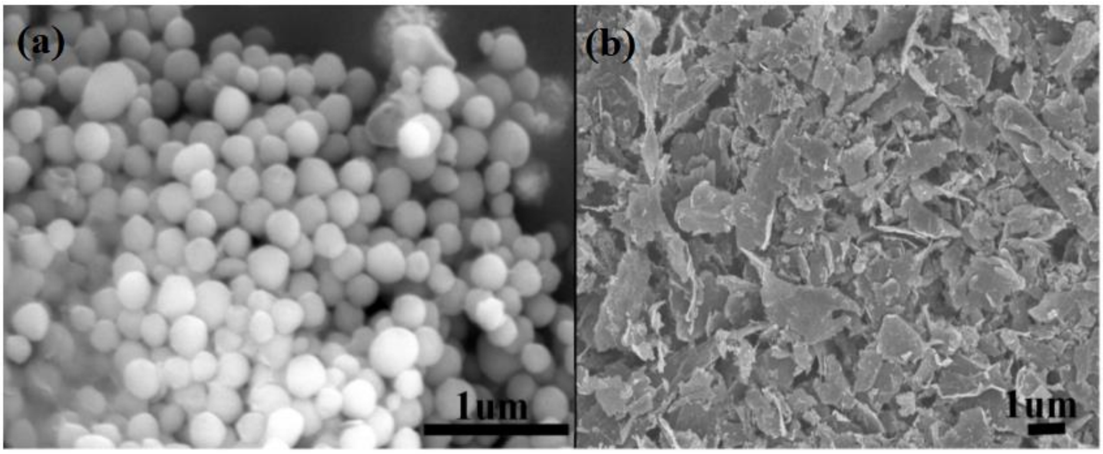

Figure S1. SEM images of (**a**) 0D nanoparticles and (**b**) 2D nanosheets for Te.

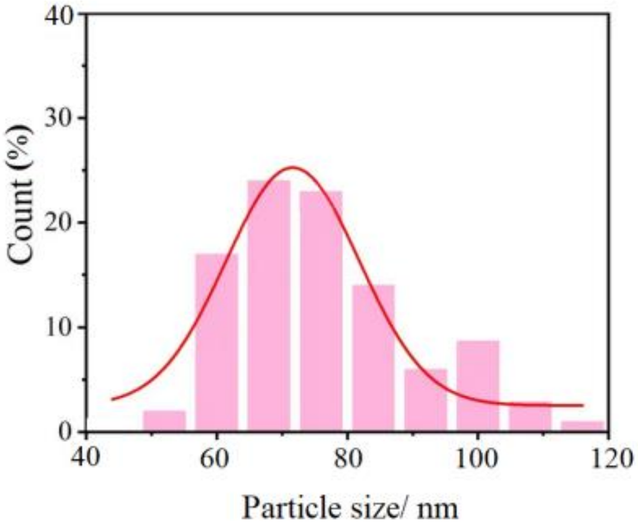

Figure S2. The particle size distribution of Te nanoparticles.

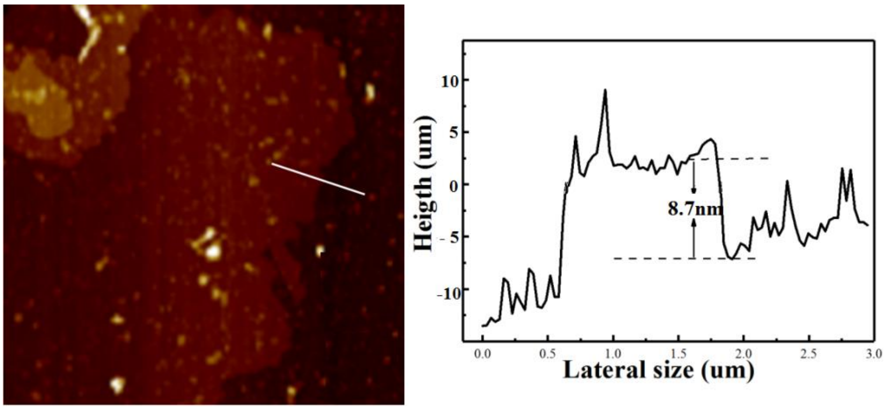

Figure S3. The thickness of Te nanosheet in AFM image.

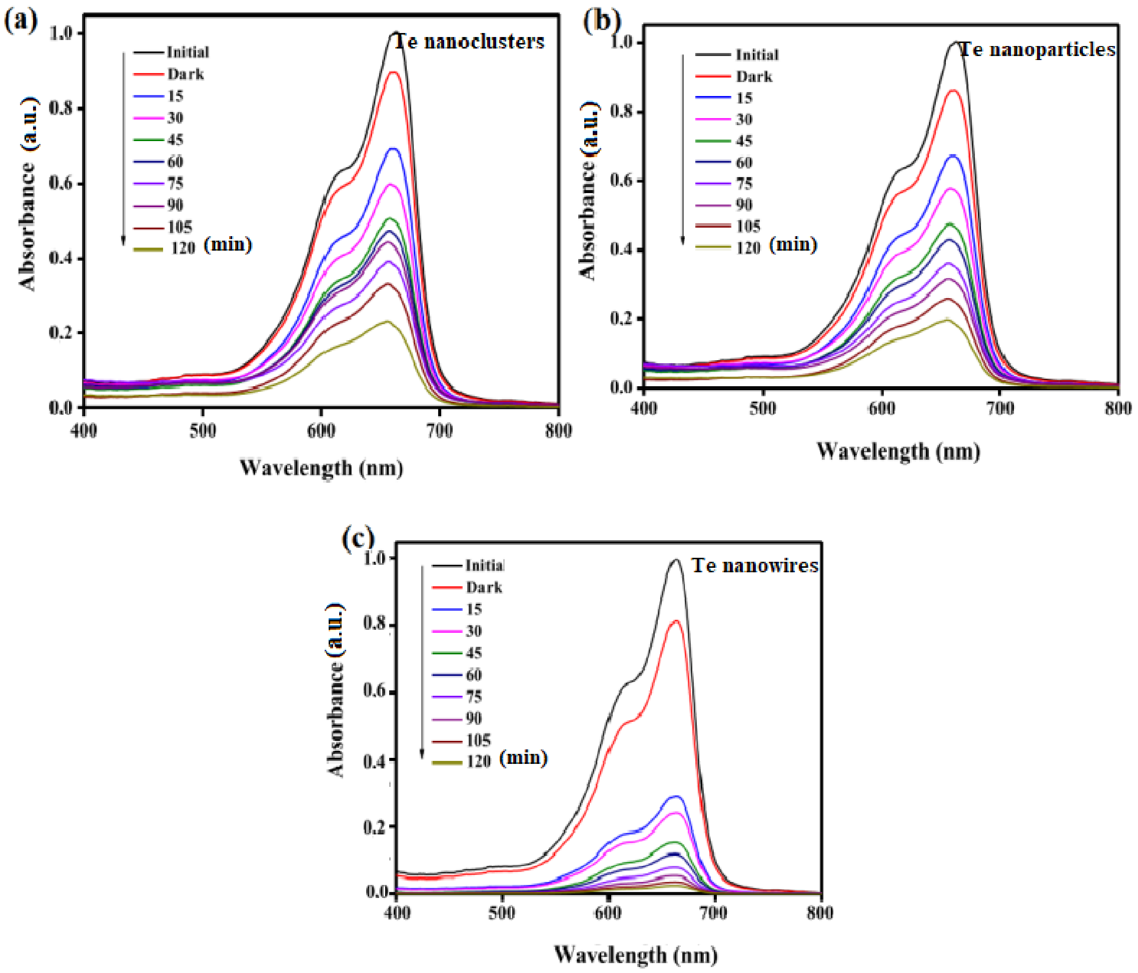

Figure S4. Time-dependent absorption spectra for MB by using (**a**) nanaoclustures, (**b**) nanoparticles, and (**c**) nanowires.

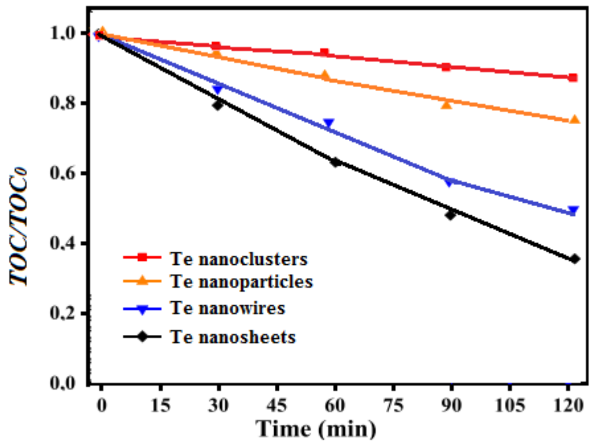

Figure S5. The TOC removal of MB with by using Te nanostructures.

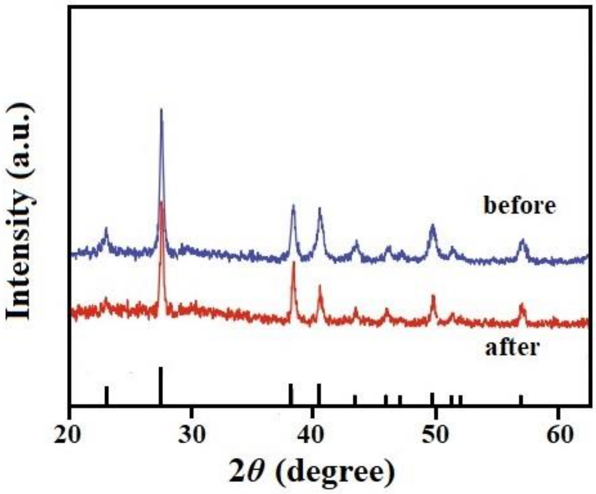

Figure S6. XRD patterns of the Te nanosheet before and after 5 cycles of photocatalytic MB degradation.
